# Conversion of Problematic Winery Waste into Valuable Substrate for Baker’s Yeast Production and Solid Biofuel: A Circular Economy Approach^§^

**DOI:** 10.17113/ftb.61.04.23.8000

**Published:** 2023-12

**Authors:** Josipa Lisičar Vukušić, Thomas Millenautzki, Leon Reichert, Abdechafik Mokhlis Saaid, Lothar Müller, Leonardo Clavijo, Jendrik Hof, Marek Mösche, Stéphan Barbe

**Affiliations:** 1 Faculty of Applied Natural Sciences, Technische Hochschule Köln, Campusplatz 1, 51379 Leverkusen, Germany; 2 Bayer Prozess Analyse Technik, Neusser Landstraße Gebäude C570, 41539 Dormagen, Germany; 3 Faculty of Engineering, Universidad de la República, Av. J. Herrera y Reissig 565, Montevideo 11300, Urugvay; 4 Uniferm GmbH & Co. KG, Industriestraße 2, 40789 Monheim am Rhein, Germany

**Keywords:** zero-waste conversion, grape pomace, solid biofuel, feedstock, baker’s yeast, circular economy

## Abstract

**Research background:**

Wine production, which is considered a major sector in food industry, often involves the use of a large amount of resources. Moreover, wine making generates a large amount of grape pomace, which is generally used for low-value applications such as fertiliser and animal feed. The aim of the present research is to explore the possibility of improving the overall sustainability of traditional winemaking.

**Experimental approach:**

A zero-waste process was developed. It includes the production of white wine and the substantial valorisation of grape pomace, which is converted into solid biofuel, tartaric acid and concentrated grape extract as feedstock for industrial baker’s yeast production.

**Results and conclusions:**

We estimate that a significant surplus of renewable energy of approx. 3 MJ/kg processed grapes can be obtained during this conversion. The suitability of grape extract as a potential substrate for industrial baker’s yeast production was evaluated and the feasibility of a partial replacement of molasses (up to 30 %) was demonstrated.

**Novelty and scientific contribution:**

We present a circular economy approach for the conversion of winery biowaste into high-value resources such as feedstock and solid biofuel.

## INTRODUCTION

**Fig. S1:**
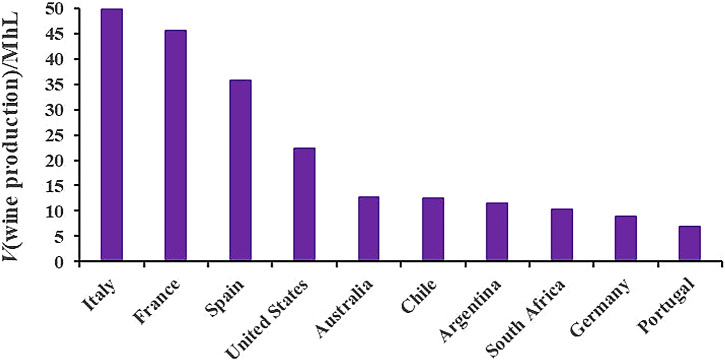
Wine production worldwide in 2022 (in million hectolitres) (*4*)

Grape (*Vitis vinifera*) is one of the most important crops and it is cultivated all over the world. This fruit is consumed both fresh and processed (mainly into wine) (*1*). In the year 2022, the world production of wine was estimated at 258 million hectolitres (*2*). Italy, France and Spain account for 53 % of the world wine production (Fig. S1) (*3*, *4*). Consequently, wine production is regarded as one of the major food industries but it involves the use of a large number of resources (water, organic and inorganic fertilisers). Wine production produces high amounts of grape pomace, which is generally used for low-value applications such as fertilisation and animal feed.

The production of white and red grape wine usually begins with the crushing of ripe grapes. For white wine, the crushed grapes are pressed to separate the juice, which is clarified and then fermented. For the production of red wine, maceration is carried out after crushing and before pressing. Fermentation is usually stopped by the separation of the yeast and the addition of sulphites (*5*). Grape pomace accounts for mass fraction about 20−25 % of the mass of the grapes crushed in wine making and consists mainly of peels (skins), seeds and stems (*6*). Its composition varies depending on grape variety, method of processing, environmental conditions and the ratio of skin/seeds/stem (*7*). However, the main constituents are (in %): water 60−80, cellulose 10−20, pectin 8−10, sugars 6−8 and lipids and proteins 2−4 (*8*). The mass fraction of organic matter of grape pomace is high, varying between 26 and 42 %.

Increasing agro-industrial activities and industrial manufactures have a huge impact on the environment, energy demand, carbon dioxide emissions and climate change. Therefore, the society and industrial manufactures are heading towards integrated sustainability (*9*-*13*). In order to contribute to a sustainability transformation, waste from different industries must serve as raw materials for new products and applications in the frame of a circular economy aiming at ’zero waste’ society.

In recent decades, the utilisation of grape pomace has been quite inefficient. Only 3 % of grape pomace produced is reused for animal feed, production of grappa or as waste-based compost. Due to the above-mentioned high organic content, this by-product is currently a growing environmental problem. Furthermore, the disposal of grape pomace on landfills poses problems related to the pollution of surface and groundwater, foul odour, potential spread of diseases by flies and pests, and oxygen depletion in the soil and groundwater by tannins and other compounds (*14*).

Due to the increasing development of sustainable agriculture and consumer demand for the preference of natural over synthetic products, the efforts to use grape pomace are now moving towards the production of: food additives, nutraceuticals, ingredients of functional foods/dietary supplements, medical remedies, fertilisers, animal feed, antimicrobial components, cosmetics and biomass fur biofuels (*15*). In 2018, approx. 7 million tonnes of grape pomace were disposed of (*16*). It is important to note that winemaking follows practices based on centuries of tradition. In this context, the application of new technological solutions designed to reduce the amount of biowaste, while maintaining the quality of the end product often requires a change in mentality. In Germany, fertiliser legislation has recently been updated with specific requirements for the application of pomace in viticulture including a specific analysis of total nitrogen as well as the regulation of the arable land that can be fertilised with this biowaste. Furthermore, the storage of grape pomace must not exceed a maximum period of 6 months. Any application of fertilisers must comply with the principles of good professional practice (*17*).

The present study aims to explore the possibility of introducing the integrated sustainability concept into traditional winemaking by transforming a problematic residual material to valuable resource. In particular, the high sugar mass fraction of grape pomace (up to 26.34 g/100 g white grape pomace and 8.91 g/100 g red grape pomace (*18*)) makes the disposal of this fraction critical. Grape pomace undergoes spontaneous fermentation very soon after pressing, resulting in a complex mixture of different organic metabolites. The key concept of this work is the simultaneous stabilisation of grape pomace and the extraction of solid/liquid sugar as a carbon source for industrial fermentations. The sugar is then recovered as an aqueous solution. It is important that the isolation of sugar is avoided because of its challenging economic feasibility.

For this purpose, the main by-product of wine production, grape pomace, was analysed as a feedstock for baker’s yeast production, one of the most important industrial bioprocesses. The raw materials used in this large-scale production process contribute significantly to the cost of this low-value bulk product (*9*, *19*). Molasses is used as the main raw material for the industrial baker’s yeast production. In order to produce a high-quality baker’s yeast with similar properties to commercial yeast, the mixture of beet and sugar cane molasses was used in this study. The molasses was only partly replaced by grape pomace (up to 30 %) to simulate the industrial parameters. Given the available amount of grape pomace and the demand for molasses, a higher mass fraction would not be realistic. Molasses is not only a carbon source for the yeast, but also contributes several other macro- and micronutrients to the fermentation process. Thus, to avoid nutrient imbalance due to the different composition of the grape pomace, the amount of molasses was limited. In this respect, the new solution to be introduced must be both ecologically and economically sustainable and acceptable so that the quality of the product remains unchanged.

In this work, white wine was produced and the resulting grape pomace was further processed into solid biofuel (pellets), concentrated grape extract and tartaric acid (mainly used as an acidifying agent, antioxidant, taste enhancer and chelating agent (*20*)).

## MATERIALS AND METHODS

### Production of Riesling wine

**Fig. 1:**
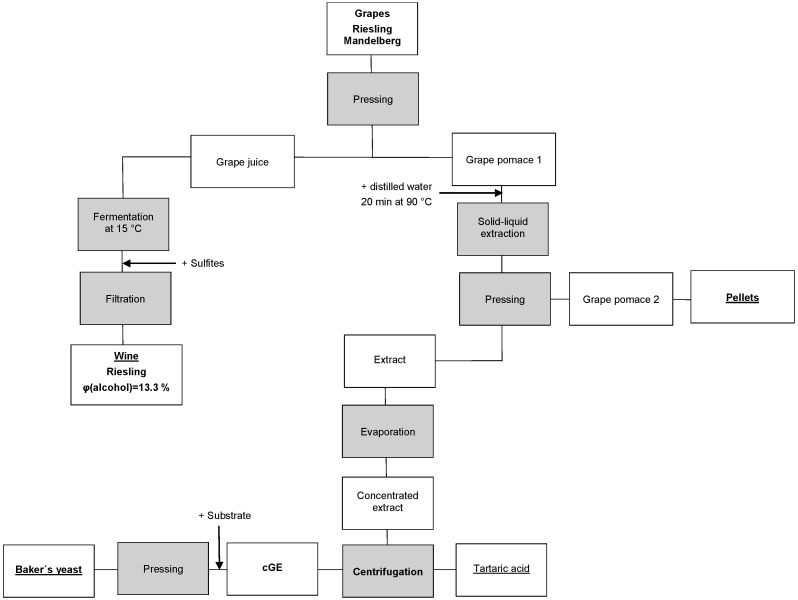
Scheme of grape processing in this research. cGE=concentrated grape extract

A mass of 84.9 kg of mature grapes (Riesling Mandelberg), kindly provided by Michael Schneider Winery (Boppard, Germany), was washed and pressed in a hydraulic press (20 L; Speidel, Ofterdingen, Germany) (Fig. 1). After pressing, 50.4 kg of grape juice and 32.5 kg of grape pomace (referred to as grape pomace 1 in the following text) were obtained. Then, the grape juice was supplemented with 10 g of mineral nutrient (VitaFerm® Ultra F3; Erbslöh, Geisenheim, Germany) and inoculated with 10 g of yeast (Oenoferm® Freddo, Erbslöh, Geisenheim, Germany) according to the producer’s instruction. The fermentation was carried out at 15 °C for 35 days and stopped by adding potassium disulfite (100 mg/L). A mass of 45.2 kg of wine was produced (5.2 kg of CO_2_ were released) and further filtered in a depth filtration apparatus (Edelstahl Weinfilter SF 10 P, Motorgeräte Fischer GmbH, Lahr, Germany) using sheet filters (20×20 FZ 20; Zambelli, Vicenza, Italy) resulting in 32.9 kg of Riesling wine with an alcohol volume fraction of 13.3 %.

### Production of grape extract and pellets

A mass of 32.5 kg of grape pomace 1 were mixed with 25 kg of deionised water, heated up to a temperature of 90 °C for 20 min under constant stirring, cooled down to 35 °C and pressed (hydraulic press 20 L; Speidel) resulting in 18.8 kg of grape pomace 2 and 28.7 kg of extract. Grape pomace 2 was dried at 60 °C for 24 h (vacuum drying oven; Heraeus Instruments, Hanau Germany) and then used as raw material for pellet production (PelletMaker PM22E; EcoWorxx GmbH, Raddestorf, Germany). Moisture content of the pellets was determined at 105 °C until constant mass. Durability was determined according to the standard ISO 17831-1:2015 method (*21*). The net calorific value of the pellets was determined according to the standard ISO 18125:2017 method (*22*).

The extract was concentrated by evaporation in a scraped surface evaporator (Normag Labor- und Prozesstechnik GmbH, Ilmenau, Germany) at a pressure of 13.4 kPa (approximate boiling temperature was 51.5 °C) and a rotation speed of 8.313*×g* to obtain 4.5 kg concentrated extract. The crystalised tartaric acid was separated by centrifugation in a Sorvall RC-5B Plus superspeed centrifuge (Thermo Fisher Scientific, Waltham, MA, USA) at 9796×*g* for 10 min resulting in 0.103 kg of tartaric acid and 3.8 kg of concentrated grape extract (Fig. 1).

The concentrated grape extract was then stored at 4 °C and its microbial stability was checked twice a week by adding a few drops of concentrated grape extract on dip slides (VWR International GmbH, Darmstadt, Germany), which were then incubated at 35 °C in a drying oven incubator (WTB Binder, Tuttlingen, Germany) over a period of three months. Furthermore, the Brix value of the extract was measured twice a week with an analogue refractometer (ORA 3SA; Kern & Sohn GmbH, Balingen, Germany).

### Preparation of media and fed-batch fermentation

The fed-batch fermentation in the production of baker’s yeast is usually initiated by inoculating a medium free of carbon source. The addition of carbon and nitrogen sources according to dosage profiles begins immediately after inoculation. In this work, fed-batch fermentations were performed with the media (carbon sources) listed in Table 1. The reference medium, molasses, was prepared as a mixture of 93 % beet and 7 % sugar cane molasses and diluted with deionised water (45 °Brix). A volume of 10 % ammonia (Bernd Kraft GmbH, Duisburg, Germany) was dosed separately as an nitrogen source and the pH value was adjusted with 25 % sulphuric acid (Merck KgaA, Darmstadt, Germany).

**Table 1:** Composition of media used for the cultivation of baker´s yeast

Medium	*w*/%		pH
Molasses	cGE
F1	100	0	5.3
F2	100	0	5.3
F3	100	0	5.3
F4	90	10	5.0
F5	90	10	5.3
F6	90	10	5.3
F7	70	30	4.5
F8	70	30	5.0
F9	70	30	5.3
F10	70	30	5.3
F11	70	30	5.3

F=fermentation, cGE=concentrated grape extract

A 1-litre bioreactor (Biostat® Qplus; Sartorius, Göttingen, Germany) was filled with 0.5 L water and 2.7 g ammonium dihydrogen phosphate (ADP; Sigma-Aldrich, Merck, St. Louis, MO, USA) and autoclaved at 121 °C for 20 min (autoclave Tuttnauer 5075 ELV; Biomedis Laborservice GmbH, Gießen, Germany). ADP is the phosphorus source and part of the nitrogen source (together with ammonia and nitrogen from molasses). Molasses and concentrated grape extract were supplemented with a trace element solution (1 mL CuSO_4_/FeCl_3_ solution per kg medium (3.52 g/L of CuSO_4_×5H_2_O and 72 g/L FeCl_3_×6H_2_O)) and 300 µL antifoam and autoclaved at 121 °C for 20 min. Molasses and concentrated grape extract were sterilised in separate flasks, except for fermentation F6, where molasses and concentrated grape extract were sterilised as a mixture. Immediately after sterilisation, the media were placed on a shaker (Shaker® KS 250; IKA, Staufen, Germany) at 150 rpm, aerated with air (3.3·10^-5^ m^3^/s) for 1 h to remove volatile, potentially inhibitory compounds such as volatile fatty acids, SO_2_ and NO_x_. The media were then supplemented with a vitamin stock: 180 μL biotin solution (1 g/L biotin and 1 g/L NaHCO_3_), 0.5 mL thiamine/pyridoxine solution (10 g/L thiamine mononitrate and 30 g/L pyridoxine hydrochloride) and 3 mL pantothenic acid solution (180 g/L calcium pantothenate). The mass loss of the medium due to water evaporation during sterilisation and aeration was adjusted with sterilised water.

**Fig. S2:**
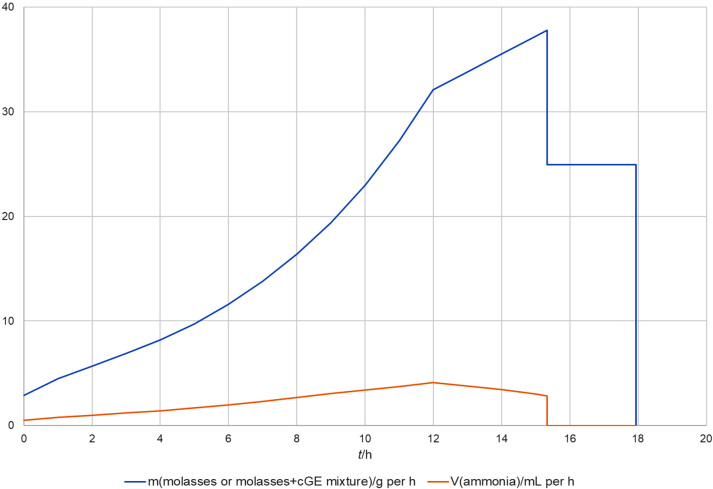
Substrate profile during fed-batch fermentation. cGE=concentrated grape extract

Substrate profiles for molasses or the mixture of molasses and concentrated grape extract, and ammonia and aeration profile were scaled down from the large-scale fermentation process. The profile of the fermentation with 100 % molasses or mixture of molasses and concentrated grape extract is shown in Fig S2. The substrate profiles of the other fermentations were very similar. The pH during sterilisation of the medium may have an effect on the stability of the sugar contained in the molasses and the concentrated grape extract. In the case of sugar degradation, this would lead to a loss of biomass yield and the formation of complex reaction products that could potentially inhibit yeast vitality and performance. After inoculation (15 to 22 g yeast, depending on the solid matter content of the corresponding inoculum taken directly from the industry), the media (molasses or the mixture of molasses and concentrated grape extract and ammonia) were gradually added according to the substrate profiles determined by Uniferm GmbH & Co. After approx. 20 h of fermentation, the bioreactor was harvested and the yeast cake was separated from the vinasse by vacuum filtration.

### Yeast characterisation

Dry matter content was determined at 140 °C for 2 h (V0101 drying cabinet; Memmert, Schwabach, Germany). Protein content was determined using the Dumas method (*23*) at 950 °C. The fermentative capacity was determined by measuring CO_2_ production in stainless steel vessels in which the pressure increase was continuously measured and recorded. The corresponding method and equipment were specially developed by Uniferm GmbH & Co. The fermentative capacity was determined in a standard dough (containing salted water and flour) and sweet dough (containing salted water, flour, butter and sugar) directly after fermentation.

## RESULTS AND DISCUSSION

### Zero waste conversion and renewable energy generation

**Fig. 2:**
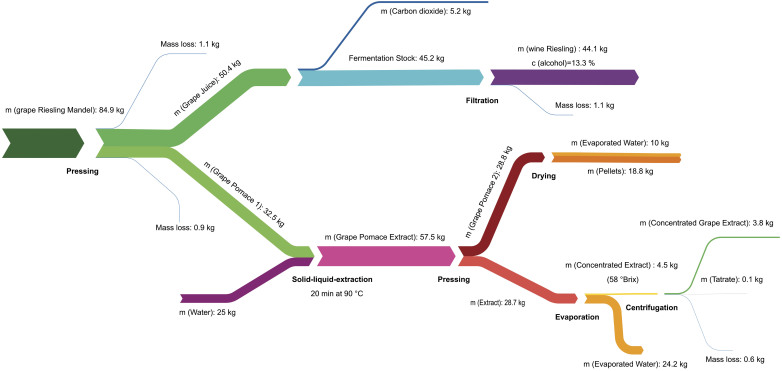
Overall mass balance during the transformation of grapes into white wine, pellets and concentrated grape extract

The mass balance of this process is shown in Fig. 2. A mass of 50.4 kg of grape juice (24 °Brix) and 32.5 kg of grape pomace (grape pomace 1 in Fig. 1) were obtained from 84.9 kg of grapes resulting in a juice yield of 59 % (Fig. 2). This value is lower than typical yields achieved in industrial winemaking (70−75 %) and may be due to the pressure limitation of our pilot scale press. The fermentation of the grape juice yielded 44.1 kg of white wine. After solid-liquid extraction, concentration and centrifugation, 3.8 kg of concentrated grape extract were obtained with a Brix value of 58 (44.8 g/kg of processed grapes). The produced grape pellets had a moisture content of 9.3 %, an acceptable durability index of 92.3 %, which is higher than that found in literature (*24*) and a net calorific value of 18.7 MJ/kg (20.6 MJ/kg based on dry matter), which is very similar to the value reported for wood pellets (*25*).

**Fig. 3:**
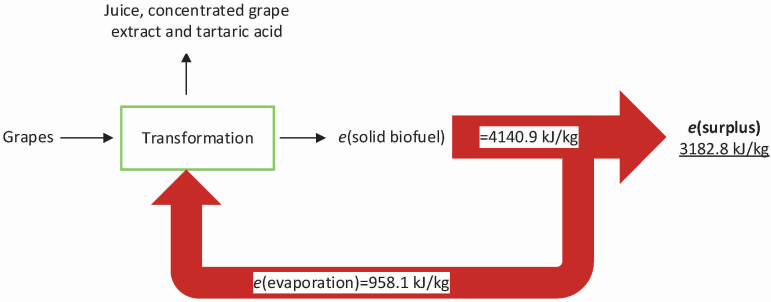
Overall balance during the transformation of grapes into juice, concentrated grape extract (cGE) and tartaric acid

Furthermore, the conversion of 1 kg of grapes generates 4140.9 kJ of energy in form of solid biofuel (0.221 kg pellets) (Fig. 3). This process requires 278.3 kJ for the evaporation of 0.118 kg of water at 60 °C (evaporation enthalpy: 2358.5 kJ/kg water (*26*)) during the drying of grape pomace 2 and 679.8 kJ for the evaporation of 0.285 kg water at 51.5 °C (evaporation enthalpy: 2385.1 kJ/kg water (*26*)) during the concentration of the grape extract. According to this heat balance, these drying steps have the major contribution to the total energy requirement of this process. Overall, we estimated that a substantial renewable energy surplus of approx. 3 MJ/kg processed grapes might be achieved during this conversion. Additionally, the vapour generated in this process can be condensed and reused for extraction and cleaning purposes.

### Quality of the produced concentrated grape extract

**Fig. S3:**
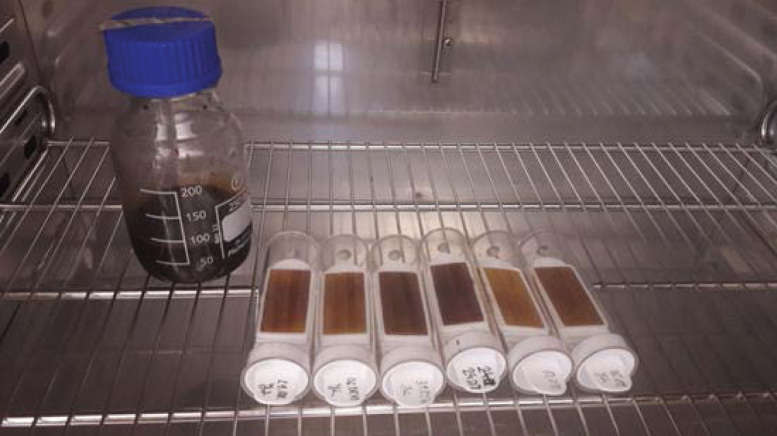
Concentrated grape extract and uncontaminated dip slides

Over a period of three months, neither a change in colour nor microbial contamination of the concentrated grape extract was observed (Fig. S3) and the Brix value remained unchanged. These observations indicate a possible long-term storage of this medium, which is a highly desirable property in an industrial environment. All fractions (wine, biofuel and concentrated grape extract) are highly valuable products and zero waste production could be achieved.

### Fed-batch fermentations

As already mentioned, the laboratory fermentation technique developed at Uniferm GmbH & Co. has been optimised over the years and the process is a scale down of the industrial fermentation process (*11*). Media containing concentrated grape extract (Table 1) were prepared in different ways to assess the potential of this alternative substrate. Sterilisation of molasses and concentrated grape extract was performed separately and together as a mixture. It was found that sterilisation did not adversely affect the concentrated grape extract, only the change of colour due to the Maillard reactions occurred (*27*). The highest amount of yeast was harvested on the media supplemented with 10 % of concentrated grape extract. The amount produced on the reference media and the media supplemented with 30 % of concentrated grape extract was the same.

**Fig. 4:**
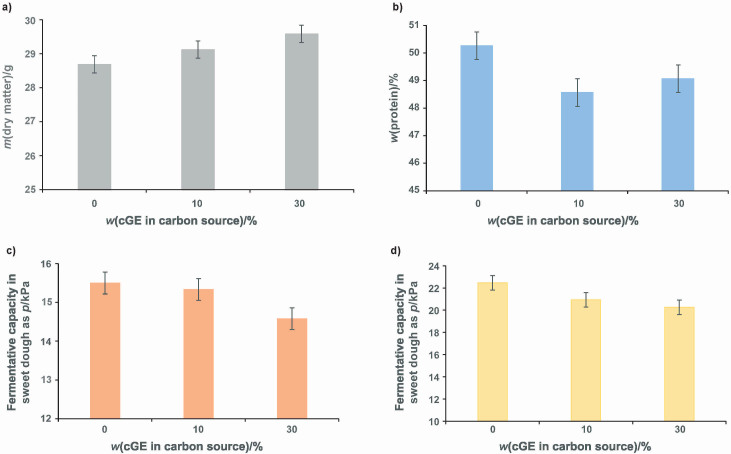
Results of the growth of baker’s yeast on different media containing molasses and concentrated grape extract: a) dry mater content of baker’s yeast, b) protein content of baker’s yeast, c) fermentative capacity of baker’s yeast tested in standard dough, d) fermentative capacity of baker’s yeast tested in sweet dough. cGE=concentrated grape extract

To evaluate the quality of the produced baker’s yeast, dry matter, protein content and fermentative capacity were measured. The results shown are the average value of the experiments (from Table 1: F1-F3 correspond to 0 % concentrated grape extract in carbon source, F4-F6 correspond to 10 % concentrated grape extract in carbon source, F7-F11 correspond to 30 % concentrated grape extract in carbon source). Fig. 4a shows the results of dry matter content of yeast grown on molasses and media containing molasses and concentrated grape extract. Dry matter content of yeast grown on molasses and concentrated grape extract is higher than the one grown on molasses only. Dry matter content of yeast produced on all media (including the reference ones containing only molasses) was according to the literature (*28*, *29*). High-quality baker’s yeast was produced on media partly replaced by concentrated grape extract, which not only reduces the need for the volatile prices of molasses (*12*), but also qualifies grape extract as an alternative substrate in this industrial production.

Another quality parameter that was measured is the protein content of the baker’s yeast (Fig. 4b). The protein content measured in the yeast grown on reference media differs only slightly from that of the yeast grown in media supplemented with concentrated grape extract. Considering that the pH value plays an important role, the medium containing only molasses was prepared with the optimal pH value for the growth (*30*). In other media, different pH values were tested. It seems that the measured protein content was higher in the yeast that was grown in the media supplemented with concentrated grape extract prepared at lower pH value (Table 1). Furthermore, it is known that the nitrogen starvation can cause decrease in the protein content of the yeast cells (*31*), so it could also explain the lower protein content of the produced yeast. Different volumes of ammonia were used in this study (Fig. S2), although different volumes have also been tested in previous studies. However, fresh baker’s yeast contains 40.6–58.0 % protein (*32*), suggesting that yeast grown on media containing both molasses and concentrated grape extract is of high quality.

One of the most important parameters of industrial baker’s yeast is the fermentative capacity: the ability to produce carbon dioxide when added to the dough (*33*). In this study, the fermentative capacity was measured in two different types of dough, standard and sweet dough (Fig. 4c and Fig. 4d). Overall, the yeast grown on reference media showed higher fermentative capacity in both types of dough (15.5 kPa in standard dough and 22.4 kPa in sweet dough). However, the fermentative capacity of the baker’s yeast grown on media supplemented with 10 % concentrated grape extract was almost the same in standard dough (15.3 kPa) and lower in sweet dough (20.9 kPa). When higher amounts of concentrated grape extract media were used in the fermentation, the fermentative capacity was lower (14.6 kPa in standard dough and 20.3 kPa for sweet dough). The yeast produced with up to 30 % concentrated grape extract can be regarded as good quality yeast. However, the negative trend of lower fermentative capacity indicates that higher amount probably has a negative effect on quality.

The results of the investigated maltose-fermenting baker’s yeast strains show poor fermentative activity in the dough with high sugar content. This could be related to the co-location of the MAL loci and the invertase (SUC) genes at the yeast telomeres. As a result, yeast strains that have duplications of the MAL loci may also have duplications of the invertase (SUC) genes, leading to overexpression of the invertase enzyme. It is also known that intrinsic osmotolerance can also be a limiting factor for the successful fermentation of yeasts in high-sugar environments. One possible explanation for why the leavening capacity of yeast decreases with increasing concentrated grape extract is the influence of the medium containing the concentrated grape extract on the expression of the MAL loci and invertase genes as well as the invertase production (*34*).

In earlier studies, the growth of baker’s yeast in grape juice was analysed. Mahmood *et al*. (*35*) reported the maximum growth of yeast biomass of 41 g/L. In addition, Lo Curto *et al.* (*36*) reported that the yields of baker’s yeast on grape marc were lower than those obtained in industrial production. However, yeast quality (especially fibre, ash and digestibility) was similar to a commercial product produced on molasses. The production of the *Saccharomyces cerevisiae* biomass was investigated on the medium with date extract as the only carbon source. Cultivation of baker’s yeast in Erlenmeyer flasks yielded 40 g/L (*37*). Agro-industrial residues of apple pomace in the production of baker’s yeast have also been investigated. Bhushan and Joshi (*38*) reported that the yield obtained with apple pomace extract was 96 % of the expected theoretical yield, thus providing an alternative to molasses. Furthermore, Joshi and Bhushan (*39*) reported that supplementation of the apple pomace extract with growth stimulators is not required for the fermentation of baker’s yeast. Lisičar Vukušić *et al.* (*13*) also reported on the potential of apple pomace as a carbon source for the growth of baker’s yeast. In the medium containing a mixture of apple pomace and molasses, a slightly lower biomass yield was obtained than in the medium containing only molasses. However, the yeast produced on the alternative substrate showed slightly higher fermentative activity than the yeast produced on molasses.

### Circular economy concept

**Fig. 5:**
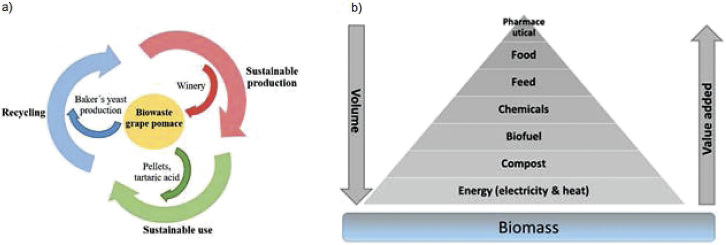
Conversion of winery waste into valuable substrate: a) circular economy approach (adapted from (*40*)) and b) biomass value pyramid (*41*)

The main outcome of this research is a circular economy concept that provides a solution for the conversion of grape pomace into a source of valuable products such as solid biofuel and a feedstock for the production of baker’s yeast (Fig. 5a) (*40*, *41*). The use of biomass as an energy source is at the bottom of the value pyramid (Fig. 5b), meaning it can be used for energy production only after higher-value uses have been fulfilled. This research therefore opens very interesting opportunities for winegrowers who try to utilise winery waste and make production more sustainable.

## CONCLUSIONS

This research shows how agro-industrial waste can be fully valorised. In this regard, high-quality yeast was produced on media that were partly replaced by concentrated grape extract. Further investigation is needed to determine the optimal ratio of molasses and concentrated grape extract as well as the optimal growth conditions for baker’s yeast. Furthermore, it was estimated that a surplus of renewable energy of approx. 3 MJ/kg processed grapes can be achieved with this conversion. The value of the grape pomace has been increased through a conversion based on a zero-waste bioprocess.
